# Beyond Antimicrobial Resistance: Evidence for a Distinct Role of the AcrD Efflux Pump in *Salmonella* Biology

**DOI:** 10.1128/mBio.01916-16

**Published:** 2016-11-22

**Authors:** Michelle M. C. Buckner, Jessica M. A. Blair, Roberto M. La Ragione, Jane Newcombe, Daniel J. Dwyer, Alasdair Ivens, Laura J. V. Piddock

**Affiliations:** a Antimicrobials Research Group, Institute of Microbiology and Infection, College of Medical and Dental Sciences, The University of Birmingham, Edgbaston, Birmingham, United Kingdom; b School of Veterinary Medicine, Faculty of Health and Medical Sciences, University of Surrey, Guildford, Surrey, United Kingdom; c School of Biosciences and Medicine, Faculty of Health and Medical Sciences, University of Surrey, Guildford, Surrey, United Kingdom; d Department of Cell Biology and Molecular Genetics, Institute for Physical Science and Technology, Department of Bioengineering, Maryland Pathogen Research Institute, University of Maryland, College Park, Maryland, USA; e Centre for Immunity, Infection and Evolution, University of Edinburgh, Edinburgh, Scotland

## Abstract

For over 20 years, bacterial multidrug resistance (MDR) efflux pumps have been studied because of their impact on resistance to antimicrobials. However, critical questions remain, including why produce efflux pumps under non-antimicrobial treatment conditions, and why have multiple pumps if their only purpose is antimicrobial efflux? *Salmonella* spp. possess five efflux pump families, including the resistance-nodulation-division (RND) efflux pumps. Notably, the RND efflux pump AcrD has a unique substrate profile, distinct from other *Salmonella* efflux pumps. Here we show that inactivation of *acrD* results in a profoundly altered transcriptome and modulation of pathways integral to *Salmonella* biology. The most significant transcriptome changes were central metabolism related, with additional changes observed in pathogenicity, environmental sensing, and stress response pathway expression. The extent of tricarboxylic acid cycle and fumarate metabolism expression changes led us to hypothesize that *acrD* inactivation may result in motility defects due to perturbation of metabolite concentrations, such as fumarate, for which a role in motility has been established. Despite minimal detectable changes in flagellar gene expression, we found that an *acrD* mutant *Salmonella enterica* serovar Typhimurium isolate was significantly impaired for swarming motility, which was restored by addition of fumarate. The *acrD* mutant outcompeted the wild type in fitness experiments. The results of these diverse experiments provide strong evidence that the AcrD efflux pump is not simply a redundant system providing response resilience, but also has distinct physiological functions. Together, these data indicate that the AcrD efflux pump has a significant and previously underappreciated impact on bacterial biology, despite only minor perturbations of antibiotic resistance profiles.

## IMPORTANCE

Efflux pumps in Gram-negative bacteria are studied because of their important contributions to antimicrobial resistance. However, the role of these pumps in bacterial biology has remained surprisingly elusive. Here, we provide evidence that loss of the AcrD efflux pump significantly impacts the physiology of *Salmonella enterica* serovar Typhimurium. Inactivation of *acrD* led to changes in the expression of 403 genes involved in fundamental processes, including basic metabolism, virulence, and stress responses. Pathways such as these allow *Salmonella* to grow, survive in the environment, and cause disease. Indeed, our data show that the *acrD* mutant is more fit than wild-type *Salmonella* under standard lab conditions. We hypothesized that inactivation of *acrD* would alter levels of bacterial metabolites, impacting traits such as swarming motility. We demonstrated this by exogenous addition of the metabolite fumarate, which partially restored the *acrD* mutant’s swarming defect. This work extends our understanding of the role of bacterial efflux pumps.

## INTRODUCTION

Resistance-nodulation-division (RND) efflux pumps in Gram-negative bacteria confer intrinsic multidrug resistance (MDR) by exporting a broad range of antimicrobial compounds out of the bacterial cell. Traditionally, *Enterobacteriaceae* such as *Escherichia coli* and *Salmonella* have five families of efflux pumps, the ABC and MFS superfamilies and the SMR, MATE, and RND families (1). Recently, the PACE family of efflux pumps was discovered in *Acinetobacter* species and is present in a range of *Proteobacteria* (2, 3). *Salmonella enterica* serovar Typhimurium (here, *Salmonella* Typhimurium) is used extensively as a model pathogen for many reasons, including easy genetic manipulation, good infection models, and relevance to other pathogens (4, 5). *Salmonella* Typhimurium has five RND MDR efflux systems: AcrAB, AcrAD, AcrEF, MdtABC, and MdsABC (5, 6). AcrB, and its homologues in other Gram-negative pathogens (e.g., MexB in *Pseudomonas aeruginosa* and CmeB in *Campylobacter jejuni*) is considered the most important RND system to human health because it transports a wide range of structurally varied antimicrobials and is more abundant within the cell than other efflux pumps (for a review, see reference 7). Furthermore, inactivation of *acrB* in *Salmonella* (and its homologues in other bacteria) confers multidrug hypersusceptibility, while single deletions of the other RND efflux pump genes have little or no effect on susceptibility to most antimicrobial agents (5, 8, 9). Interestingly, AcrD has 70% nucleotide and 79% amino acid similarity with AcrB (9), yet it possesses a distinct substrate profile that includes aminoglycoside antibiotics (10, 11). AcrB and AcrD also differ in the structures of their proximal binding pockets, which may underpin the differences in their substrate profiles (12). We have previously shown that the AcrD efflux pump impacts biofilm formation, as evidenced by an *acrD* mutant that showed significantly reduced biofilm formation and reduced expression of key biofilm proteins encoded by *csgBD* (13).

Little is known about the natural and potentially homeostatic functions of efflux pumps in pathogen biology. Efflux pumps are evolutionarily ancient proteins which long predate the use of antibiotics (14). In *Salmonella*, the AcrAB-TolC efflux pump is involved in protection from and efflux of bile and toxic bile salts (15–21). In *E. coli*, a physiological substrate is the siderophore enterobactin, which is effluxed by AcrAB-TolC, AcrAD-TolC, and MdtABC pumps (22–24). To further understand the natural function of AcrB in *Salmonella*, we previously determined the transcriptome of a *Salmonella acrB* mutant; the effect was profound and correlated with an altered basic biology of the organism (25). Furthermore, we previously showed that inactivation of individual or multiple *acr* efflux pump genes results in increased expression of the remaining efflux pump genes, suggesting a sensing mechanism to detect and regulate relative expression levels of the different pumps (9). When *acrB* was deleted, *acrD* expression increased (9). While the physiological role of AcrB is becoming clearer, much less is known about the functions of AcrD. Therefore, we sought to understand how loss of AcrD impacted the biology of the organism and whether loss of this pump led to changes to *Salmonella* physiology.

In the current study, we determined the transcriptome of a *Salmonella* mutant, constructed and tested previously (8, 9), in which *acrD* had been genetically inactivated. Here, we demonstrate that multiple genes, including those involved in metabolism, stress responses, and virulence, were altered by *acrD* inactivation. Using these transcriptomic data as a guide, we examined phenotypic differences between the parental strain and the *acrD* mutant. Specifically, we explored the ability of an *acrD Salmonella* mutant to swim and swarm, grow on various carbon sources, grow under anaerobic conditions, and invade polarized epithelial cells, and we tested the fitness of the *acrD* mutant relative to the parental strain. Our data suggest that AcrD has a different role in the biology of the organism than previously assumed.

## RESULTS

### Inactivation of *acrD* leads to distinct transcriptional changes.

**FIG 1:**
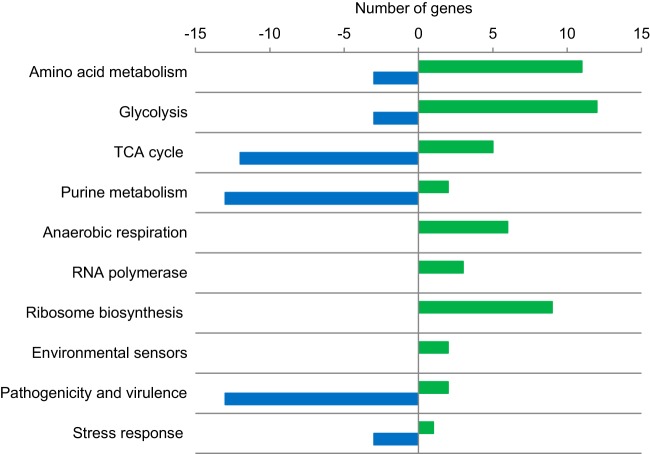
The gene expression changes of SL1344 *acrD*::*aph* compared to wild-type SL1344. Genes with altered expression in the *acrD* mutant were categorized according to function. The horizontal axis indicates the number of genes in each category, with negative values indicating genes that were downregulated and positive values indicating genes that were upregulated.

The transcriptome of the *acrD* mutant revealed significantly altered expression of 403 genes compared to the parental SL1344 strain (for a list of genes with significantly altered expression, see Table S1 in the supplemental material; full microarray data are available at http://www.ebi.ac.uk/arrayexpress/experiments/E-MEXP-2975/samples/). Specifically, in the *acrD* mutant expression of genes encoding proteins involved in anaerobic growth, ATP synthesis, amino acid metabolism, sugar transport, glycolysis/gluconeogenesis, ribosomal subunit biosynthesis, RNA polymerase, and oxidative phosphorylation were generally increased (Fig. 1). Expression of genes associated with pathogenicity, sigma factors, stress response, the tricarboxylic acid (TCA) cycle, and purine metabolism were generally decreased (Fig. 1). Surprisingly, very few of the transcriptomic changes could be directly linked to drug export.

It seemed unlikely that the changes observed at the transcriptomic level were simply due to the lack of a large membrane protein. If this were the case, we would expect the deletion of *acrD* and *acrB* to result in similar changes and also changes in membrane stress response genes, e.g., *cpxAR* and *baeSR* (26–28). Both CpxR and BaeR are known to induce expression of efflux pumps, including AcrD (26, 29, 30). In *E. coli*, inactivation of *tolC* activates the Cpx and Bae pathways (28). However, there was no significant change in expression of either the *cpx* or *bae* gene in the *acrD* mutant.

### Carbon metabolism is largely unaltered by loss of *acrD*.

Since many of the observed transcriptional changes involved genes that encode products involved in carbon utilization, the Biolog phenotypic microarray system (Biolog, Inc., USA) was used to measure the respiration of the wild type and *acrD* mutant on various carbon sources (PM1 and PM2A). The respiration levels on different carbon sources for the *acrD* mutant and the wild-type strain were very similar, with only one major difference observed. The *acrD* mutant had significantly higher respiration levels on saccharate relative to that of wild-type *Salmonella* (*P* = 0.0037).

In order to validate the results from the phenotypic microarrays and to determine if the change in respiration was also associated with a change in growth, selected carbon sources were tested. Growth in M9 minimal medium supplemented with glucose, fructose, succinate, aspartate, pyruvate, or saccharate was examined (see Fig. S1 in the supplemental material). As a control, growth of the *acrD* mutant and wild-type *Salmonella* in LB broth was measured, and no differences were found in generation time or the final optical density (OD) at 19 h (Fig. S2). The generation time of the *acrD* mutant was not significantly different when grown with glucose, fructose, succinate, aspartate, pyruvate, or saccharate as a carbon source (Fig. S3a). Likewise, measurement of the final OD of the culture indicated that the *acrD* mutant and wild-type strain grew to similar final densities on the carbon sources tested (Fig. S3b).

### Inactivation of *acrD* leads to increased expression of nitrate reductase and nitrite reductase genes.

In the *acrD* mutant, the expression of six genes (*napACF* and *nirBCD*) involved in anaerobic growth was increased between 3.06- and 22.83-fold (Table S1). However, under anaerobic conditions, there was no significant difference in the generation time (Fig. S4a) or the final OD (Fig. S4b) of the *acrD* mutant culture compared to the wild-type parental strain. Additionally, there was no difference in growth in 1 mM sodium nitrate, nor was there a difference in survival in 10 mM acidified sodium nitrite (data not shown).

### Inactivation of *acrD* results in reduced virulence gene expression.

Inactivation of *acrD* led to decreased expression of 13 genes known to be involved in *Salmonella* virulence. The expression levels of genes from *Salmonella* pathogenicity islands 1 (SPI-1), -2, -3, -10, and -18 were significantly decreased (0.05- to 0.5-fold, which corresponds to a 20- to 2-fold reduction) (see Table S1). This included reduced expression of genes encoding secreted proteins (*cigR* and *sseBCDE*) and virulence protein chaperones (*sicP* and *sscAB*), while expression of a gene encoding a needle apparatus sorting platform component (*orgA*) was increased.

**FIG 2:**
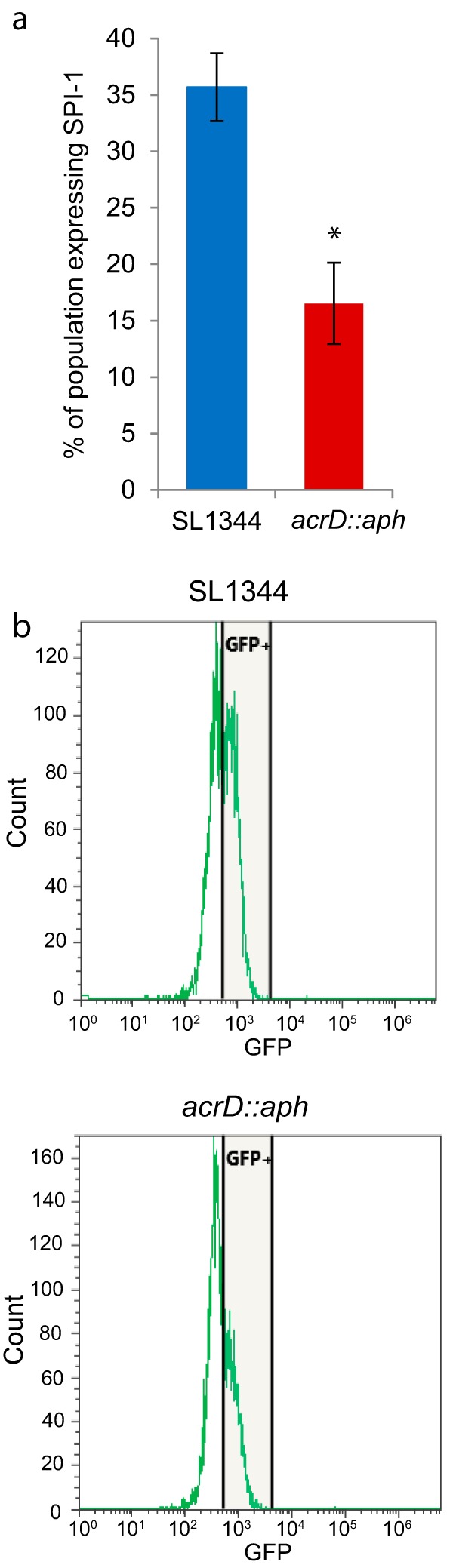
SPI-1 expression of the *acrD*::*aph* strain. Strains containing the promoter of *prgH* (SPI-1) fused to *gfp* were analyzed by flow cytometry and the number of fluorescent and nonfluorescent cells were enumerated. (a) Mean results ± standard deviations. *, *P* < 0.05. (b) A representative example of the flow cytometry results for SL1344 and SL1344 *acrD*::*aph* cells.

Genes from SPI-1 have been shown to be expressed by only a portion of the total population (31–33), so techniques such as microarrays and reverse transcription-PCR (RT-PCR), which measure gene expression across a whole population, only provide partial information about gene expression of individual bacteria in a population. The expression of the SPI-1 gene *prgH*, which encodes the essential basal component of the needle complex of the SPI-1 type III secretion system, has been used previously to measure expression of SPI-1 in single cells (31, 34). Therefore, strains containing the promoter of *prgH* fused to the green fluorescent protein gene *gfp* were used to compare the percentage of cells in the population that expressed SPI-1 in the *acrD* mutant versus in the wild type, as described previously (31, 34). This showed that when *acrD* was inactivated, fewer cells expressed SPI-1 than did wild-type *Salmonella* cells (Fig. 2). These findings correlate with the decreased detection of SPI-1 transcripts in the *acrD* mutant microarray analysis (Table S1).

Intriguingly, this reduction in the proportion of bacteria expressing SPI-1 did not correlate with decreased invasion of the *acrD* mutant into Caco-2 polarized epithelial cells. There was no difference in the virulence of the *acrD* mutant compared to the parental SL1344 strain; as there was no difference between wild-type SL1344 and the *acrD*::*aph* strain in association (2 h), invasion (4 h), and persistence (8 and 24 h) in Caco-2 polarized epithelial cells (*P* = 0.745, *P* = 0.846, *P* = 0.942, and *P* = 0.685, respectively) (Fig. S5).

### Inactivation of *acrD* reduces motility.

**FIG 3:**
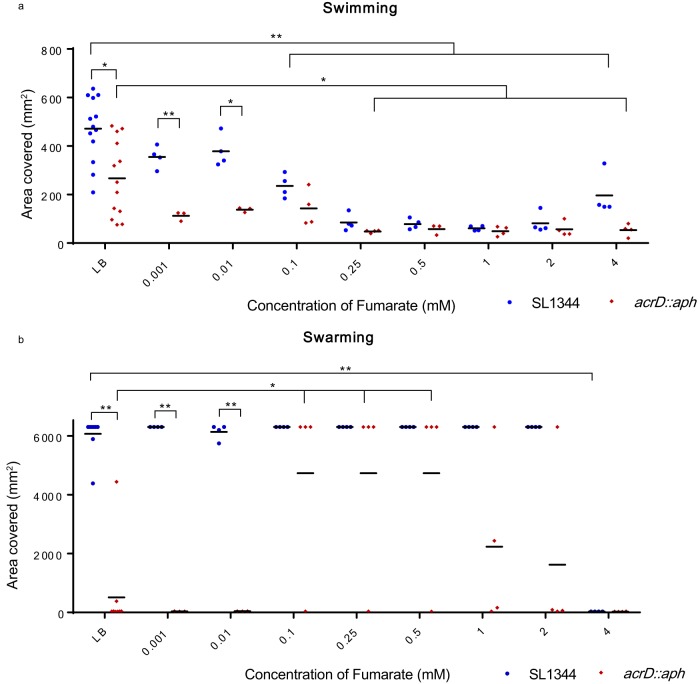
Impact of fumarate on motility of *acrD*::*aph* mutant cells compared to SL1344 cells. Areas covered by SL1344 and SL1344 *acrD*::*aph* mutant cells for swimming (a) and swarming (b) motility in LB alone and in the presence of increasing concentrations of fumarate are summarized. Data presented are from 13 replicates for LB alone and 4 replicates for fumarate, with mean values indicated. *, *P* < 0.05; **, *P* < 0.001.

Compared to SL1344, the *acrD* mutant was impaired for swimming. SL1344 swam to cover an average area of 471.5 mm^2^, while the *acrD* mutant covered 266.7 mm^2^ (*P* = 0.0018) (Fig. 3a). Likewise, the *acrD* mutant was impaired for swarming compared with SL1344, which covered an average area of 6,090 mm^2^ while the *acrD* mutant covered 510 mm^2^ (*P* < 0.001) (Fig. 3b). The swimming assay produces results that are inherently variable, and so we minimized this impact by performing 13 replicates.

Since there were no changes in expression of common motility genes found in the transcriptome (e.g., flagellar genes), yet we observed a drastic impact on motility, we explored the effect of metabolites on motility. This was because expression levels of many metabolism-associated genes were altered in the transcriptome. Fumarate has been shown to impact the switching of the direction of flagellum rotation (35–37). Furthermore, the transcriptomic data showed increased expression of *frdABCD* and *aspA*, as well as decreased expression of *sdhCDA* and *fumC* (see Table S1), the gene products of which impact fumarate levels. To determine if fumarate could restore the motility defect seen in the *acrD* mutant, exogenous fumarate was added to the culture medium. At concentrations of 0.1, 0.25, 0.5, 1, 2, and 4 mM, fumarate inhibited swimming motility of wild-type SL1344 (*P* = 0.004, *P* < 0.001, *P* < 0.001, *P* < 0.001, *P* < 0.001, *P* = 0.0016, respectively), while concentrations of 0.01 and lower did not impact swimming (Fig. 3a). For the *acrD* mutant, concentrations of 0.25, 0.5, 1, 2, and 4 mM fumarate inhibited swimming motility (*P* = 0.015, *P* = 0.041, *P* = 0.016, *P* = 0.020, *P* = 0.018, respectively) (Fig. 3a). Fumarate concentrations between 0.1 and 0.5 mM increased the swarming ability of the *acrD* mutant (*P* < 0.05 for 0.1, 0.25, and 0.5 mM fumarate) (Fig. 3b) compared with LB alone. Our data indicate that the ability to swarm is either on or off. In LB alone, 9 replicates showed that the wild type swarmed very well and only one replicate showed moderate swarming; for the *acrD* mutant, 8 replicates did not swarm at all and 2 replicates swarmed moderately (Fig. 3b). At concentrations of 0.1, 0.25, and 0.5 mM fumarate, the proportion of *acrD* mutants which did swarm increased (*P* < 0.05) (Fig. 3b). At 4 mM fumarate, swarming of the wild type was inhibited (*P* < 0.001) (Fig. 3b). One limitation of this experiment was that the swarming wild-type bacteria reached the edge of the plate, and we were unable to determine if fumarate increased swarming of SL1344 by the same magnitude as in the mutant.

### Inactivation of *acrD* impacts bacterial fitness.

**FIG 4:**
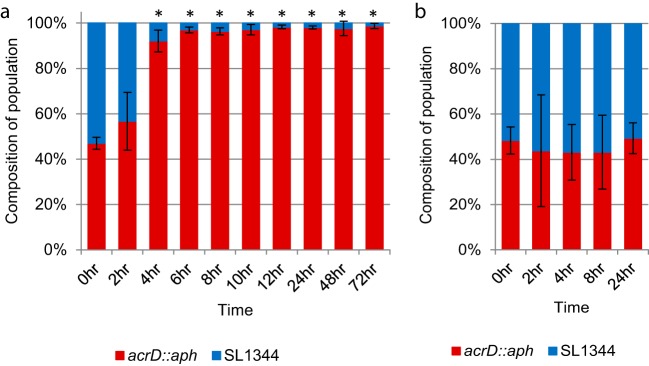
Impact of *acrD* inactivation on bacterial fitness. (a) Fitness under standard laboratory conditions *in vitro*. LB broth was inoculated with a 50:50 mixture of wild-type and *acrD* mutant cells and grown at 37°C with aeration. Samples were taken at the indicated time points, and fresh cultures were inoculated every 24 h. Data presented are the means of three independent experiments with three biological replicates each, ± standard deviations. (b) Competitive infection of polarized Caco-2 epithelial cells. After infection with a 50:50 mixture of wild-type and *acrD* mutant cells, association was determined at 2 h postinfection prior, to gentamicin treatment. A gentamicin protection assay was used to calculate invasion and persistence at later time points. Replica plating was used to determine the proportion of wild-type versus *acrD*::*aph* bacteria. Data presented are the means of three independent experiments performed, with 4 replicates each, ± standard deviations. *, *P* < 0.05; **, *P* < 0.001.

Since no differences were seen in growth when evaluated under a variety of conditions, yet expression levels of 403 genes were altered, we hypothesized that these numerous gene expression changes would alter the fitness of the *acrD* mutant in a competitive environment. Bacterial fitness can be determined in a number of different ways; initially we tested fitness under standard laboratory conditions *in vitro* (LB broth, 37°C with aeration, inoculation with a 50:50 ratio of wild-type *Salmonella* to *acrD* mutant). Under these conditions, the *acrD* mutant outcompeted the wild type as early as 4 h postinoculation (Fig. 4a). After 2 h of growth, the *acrD* mutant composed 57% of the population, and by 4 h 92% of the bacteria were *acrD* mutants (*P* < 0.001). This high proportion rose to 97% at 6 h (*P* < 0.001) and then varied between 96% and 99% (*P* < 0.001) for the remainder of the 72 h, which included passaging into fresh medium at 12, 24, and 48 h.

To measure fitness under *in vivo* conditions, competitive infection experiments were carried out. Polarized Caco-2 epithelial cells were infected with a 50:50 ratio of wild-type *Salmonella* to the *acrD* mutant. At 2, 4, 8, and 24 h postinfection, the proportion of the *acrD* mutant to the wild-type SL1344 was not significantly altered (Fig. 4b). This indicated there was no fitness differential associated with the loss of *acrD* during the infection of polarized Caco-2 epithelial cells (Fig. 4b).

## DISCUSSION

Our data suggest that the AcrD efflux pump has a unique biological role. This was demonstrated by the significant changes observed in the transcriptome. Comparison of the transcriptomes of the *acrD* mutant (presented in this work; further information is available at http://www.ebi.ac.uk/arrayexpress/experiments/E-MEXP-2975/samples/) with the previously published *acrB* mutant transcriptome (25) showed that the effect was very distinct, which supports the hypothesis that AcrD is not a “backup” efflux pump but has its own physiological purpose in the cell. This comparison identified 232 significant gene expression changes specific to the inactivation of *acrD* and that were not affected by the disruption of *acrB*. Compared to the *acrB* mutant transcriptome, 169 genes were differentially expressed by both *acrB* and *acrD* mutants. Of these, expression of 91 genes was altered in the same way (e.g., increased in both mutants), and 78 genes were expressed in an opposite manner (e.g., increased in one but decreased in the other). There is experimental evidence that AcrB and AcrD efflux pumps have distinctive substrate profiles with respect to aminoglycoside antibiotics (10–12). Our data showed that deletion of either *acrB* or *acrD* has different impacts on the bacterial transcriptome, which supports the suggestion that these pumps have distinct roles. We speculate that accumulation of certain metabolic intermediates in the bacterial cell may trigger feedback mechanisms which alter gene expression and metabolism in different ways (23, 38).

Some of the genes identified in the microarray were drastically altered in the *acrD* mutant; for example, *adh* expression increased 70-fold and *fruB* increased 45-fold. On the other hand, some genes had <10-fold changes but these changes were still statistically significant. These data are included in Table S1 in the supplemental material for several reasons. First, depending on gene function, small changes in expression can translate into significant changes in the biology of the organism (39, 40). Second, multiple small changes which occur in the same or related pathways can have strong impacts on phenotypes (41, 42). In the data presented here, the expression changes in genes involved in fumarate metabolism (*frdABCD* and *sdhCDA*) were below 10-fold, yet fumarate had a direct and significant impact on the *acrD* mutant’s motility (Fig. 3). Third, these data, including the large and small changes, will likely prove useful for the scientific community.

Nearly 100 genes associated with metabolism were altered by the inactivation of *acrD*. Table S1 lists genes with significantly altered expression; selected genes and operons are discussed below. Expression of the pyruvate formate lyase I gene, *pflB*, increased. PflB is involved in glucose metabolism, converting pyruvate to formate and acetate, and is preferentially used under anaerobic conditions (43, 44). Abernathy et al. (44) found that deletion of *pflB* led to increased intracellular replication in intestinal epithelial cells as a result of increased SPI-1 expression. In our study, the *acrD* mutant had increased *pflB* expression and reduced SPI-1 expression, indicating another connection between efflux pump gene expression, metabolism, and virulence. The fructose operon (*fruBKA*), under the control of FruR (45), was upregulated in the *acrD* mutant. However, there was no difference between growth of the *acrD* mutant and wild type when fructose was used as a carbon source. Another metabolism gene, *ilvC*, had higher expression in the *acrD* mutant. IlvC is a component of the pathway *Salmonella* uses to synthesize isoleucine and valine, and it is encoded by *ilvGEDAYC* (46). *ilvGEDA* is an operon, but transcription of *ilvC* is separate and dependent on IlvY (46). We were surprised that the Biolog phenotypic microarray data did not indicate significantly altered respiration of the *acrD* strain on carbon sources other than saccharate. We hypothesize that the effect of the transcriptional changes may be compensatory changes within the mutant to ameliorate the effect of loss of AcrD.

Six genes, *napACF* and *nirBCD*, associated with nitrate and nitrite reduction were upregulated when *acrD* was inactivated. This contrasts with the reduction in expression of these same genes in the *acrB* mutant (25). *Salmonella* has two nitrate reductases, one located in the cytoplasm and the other in the periplasm (NapACF) (47). NirBD is an NADH-dependent nitrite reductase located in the cytoplasm, while NirC is a nitrite/proton antiporter in the bacterial membrane, that allows transport of nitrite into the cytoplasm for detoxification (48, 49), and contributes to *Salmonella* virulence in both mice and macrophages (50). However, there was no difference in the ability of the *acrD* mutant to grow anaerobically compared to wild-type *Salmonella*, nor was there a significant difference in the ability of the mutant to grow in the presence of sodium nitrate or survive in acidified nitrite. It seems plausible that deletion of these genes, as in studies performed by other groups, has a greater effect on phenotype than the level of the increased expression (3.5- to 23-fold) seen in our *acrD* mutant.

Among the differences in the transcriptomes, the expression levels of genes involved in virulence were reduced in the *acrD* mutant. *Salmonella* has several pathogenicity islands, including SPI-1 and SPI-2, which contribute to invasion of nonphagocytic cells as well as survival and replication within host cells (4, 51). The SPI-1-associated genes, which were downregulated in the *acrD* mutant, were different from the SPI-1 genes downregulated in the *acrB* mutant (25). Our transcriptomic data indicate that inactivation of *acrD* led to reduced expression of SPI-1- and SPI-2-associated chaperones, translocon components, and effectors. Despite the reduced expression of some SPI-1-associated genes and the reduced proportion of bacteria expressing the SPI-1 apparatus, neither association, invasion, nor persistence in Caco-2 treated cells was impacted by the loss of *acrD*. A previous study also showed no impact of *acrD* inactivation on adhesion or invasion of INT-407 epithelial cells or on colonization and persistence of 1-day-old and 2-week-old chicks between the *acrD* mutant and wild-type strain SL1344 (52). In line with these findings, Nishino et al. (2006) showed inactivation of *acrD* had minimal impact on BALB/c mouse survival (5). In contradiction to the findings of Buckley et al. (52) and Nishino et al. in 2006 (5), another study found that the *acrD* strain was attenuated in an INT-407 model of infection (9). However the polarized Caco-2 epithelial cells used in our study are more physiologically relevant. Furthermore, our competitive infection data confirm that the *acrD* mutant is as fit as wild-type *Salmonella* during infection. *Salmonella* invasion of polarized epithelial cells occurs in a highly cooperative manner with multiple bacteria engulfed by *Salmonella* induced membrane ruffles (53). We hypothesize that this cooperative entry accounts for this apparent contradiction, as cells that express SPI-1 induce ruffles, which engulf both SPI-1 expressing and non- SPI-1-expressing bacteria.

The *acrD* mutant was able to outcompete the wild-type *Salmonella in vitro* in the fitness experiment in LB culture medium. We postulate that the metabolic shifts highlighted by the transcriptomic data provide an advantage to the *acrD* mutant in the LB fitness experiment. The cumulative effect of changes to metabolic gene expression does not impair the ability of the mutant to outcompete the parental strain.

*Salmonella* species are highly motile, and are capable of swimming and swarming in a flagella dependent manner (54, 55). Swimming is associated with counterclockwise rotation of the flagella, and is often interspersed with tumbles, brought about by switching direction of flagellar rotation (56). This ability to switch rotation direction is also important for swarming motility, and is controlled by the chemotaxis response regulator CheY (56). Swarming *Salmonella* move within a wet “slime” layer composed of polysaccharides, surfactants, and peptides (57, 58). In a gene expression study conducted by Wang *et al*. 2004 (57), the flagella operons were not upregulated during swarming. Swarming *Salmonella* also have distinct changes in basic metabolism, with glucose a key energy source for the process (59). We made similar observations in this study; expression of multiple metabolic but not flagella specific genes was altered in the *acrD* mutant, yet significant changes to swarming were detected.

Recent studies have focused on finding novel genes and/or pathways which mediate swarming (60-62). A screen of a mutant library containing 1023 mutants in *S. typhimurium* 14028s found 21 mutants with impaired swimming and swarming, forty nine with impaired swimming, but normal swarming, and also 49 with impaired swarming but normal swimming, and 39 hyper-motile mutants (61). Further exploration of these novel motility genes identified by Bogomolnaya *et al.* 2014 is needed. Future work uncovering more pathways and proteins directly involved in swarming may shed further light on our data.

Despite the drastic reduction in swarming motility of the *acrD* mutant, we did not see significant changes in the expression of genes known to impact motility and swarming, including *cheY*. We observed significant changes in expression of genes associated with the production of fumarate. The key enzymes in the interconversion of succinate and fumarate are FrdABCD and SdhCDAB (63). Under aerobic conditions, *sdh* is upregulated and these proteins catalyze the oxidation of succinate at higher frequency (64). In the *acrD* mutant, the *sdhCDA* genes were downregulated. Under anaerobic conditions, *frd* genes are upregulated and fumarate can act as a terminal electron acceptor; however it is a low energy yielding acceptor (63-66). In the *acrD* mutant the *frdABCD* operon was upregulated, yet no difference in anaerobic growth was seen. Together with the upregulation of *aspA* and downregulation of *fumC* we hypothesized that the *acrD* mutant has altered intracellular levels of fumarate.

Fumarate is associated with flagella rotation switching in a model which uses cytoplasm free envelopes of both *Salmonella* and *E. coli* where flagella are present, but do not normally change directions (35). However, the addition of 1 mM fumarate restores the switching of flagella, even in the absence of CheY (35, 37). With the addition of fumarate the flagella rotated in the clock-wise direction more often; fumarate was shown to target the switch motor complex (37). In line with these findings, when we added fumarate (0.001 to 4 mM) to the swarming assay medium, there was a clear increase in swarming of the *acrD* mutant at concentrations of 0.1 to 0.5 mM. The intracellular concentration of fumarate in *S. typhimurium* has previously been shown to be 0.23 µM (± 0.12) (67), thus the exogenous levels of fumarate needed to impact swarming (0.1 to 1 mM) are higher than those found within the cell. Furthermore, there was an “all-or-nothing” swarming phenotype, and the addition of fumarate increased the proportion of the mutant bacteria that swarmed. Taken together, we hypothesize that inactivation of the AcrD efflux pump leads to altered levels of fumarate, which directly impacts swarming motility.

A recent elegant study examined the localization of AcrB, AcrD and TolC within the membrane (68). In this study they found that AcrB and AcrD formed stabilized foci when bound to TolC and that as levels of AcrD increased, AcrB was displaced from TolC, a phenomenon the authors call transporter exchange (68). This exchange did not occur when AcrB substrates were present (68). Extrapolating this hypothesis provides a potential explanation of our data; when *acrD* is deleted there are fewer competitors for AcrB to bind with TolC and therefore, the AcrAB-TolC complex is stabilized, even in the absence of AcrB substrates. We postulate this could affect the metabolite concentrations within the cell.

In summary, our study demonstrates important and significant differences in bacterial gene expression occur as a result of inactivation of *acrD*, and this response is different to the response elicited by the inactivation of *acrB*. This work highlights a previously underappreciated role of AcrD in the fundamental biology of *Salmonella*.

## MATERIALS AND METHODS

### Bacterial strains and culture media.

All strains were derived from *Salmonella enterica* serovar Typhimurium SL1344 (69), and the efflux pump-inactivated mutant (SL1344 *acrD*::*aph*) has been previously described (8, 9). LB broth (Sigma-Aldrich, United Kingdom), M9 minimal medium (components from Sigma-Aldrich, United Kingdom), and morpholinopropanesulfonic acid (MOPS) minimal medium supplemented with 0.2% glucose (Teknova, USA) were used throughout this study.

### Microarrays.

Microarray experiments were carried out and results were analyzed exactly as described previously for other strains; they were carried out in parallel to those with an SL1344 *acrB*::*aph* mutant (25, 70). Briefly, overnight cultures of S. Typhimurium SL1344 and the mutant strain were grown in MOPS minimal medium (glucose) at 37°C until early logarithmic phase (OD_600_, ~0.7). For each strain, three biological and two technical replicate RNA preparations were made. Data were analyzed with the Bioconductor (71) and Pathway Tools (72) programs. Data with a B (log odds value) value of ≥0, which corresponds to an adjusted *P* value of <0.004, were considered significant. The microarray data set is available at http://www.ebi.ac.uk/arrayexpress/experiments/E-MEXP-2975/samples/ (submitted 10 November 2010, released 1 June 2011, last updated 2 May 2014; minimal medium used [http://www.ebi.ac.uk/arrayexpress/protocols/87504/?ref=E-MEXP-2975]).

### Phenotypic microarrays.

*Salmonella* Typhimurium strains were cultured from frozen (−80°C) stocks on LB agar for 24 h at 37°C, aerobically. Bacteria were harvested from agar plates and resuspended into IF-0a inoculating fluid (Biolog, Inc., USA) and adjusted to 42% transmittance (turbidimeter; Biolog). A working cell suspension of 10 ml IF-0a, 2 ml of bacterial suspension, and 120 µl of tetrazolium dye (90 µl dye D, 30 µl dye F; Biolog) was made for the PM1 and PM2A carbon utilization plates. One hundred microliters of suspension was added to each of the 96 wells on the appropriate PM plates. The inoculated PM plates were placed in an Omnilog automatic plate reader (Biolog) and incubated at 37°C, aerobically, for 48 h. Metabolism of the various carbon sources was recorded every 15 min based on the reduction of the tetrazolium violet redox dye, which produces a purple color indicative of active bacterial respiration. Abiotic negative-control plates indicated false-positive results due to autoreduction of the dye observed in PM1 with l-arabinose, d-xylose, d-ribose, or l-lyxose. False-positive results observed in PM2A included d-arabinose, 2-deoxy-d-ribose, d-glucosamine, 5-keto-d-gluconic acid, and dihyroxyacetone.

Each PM plate preparation was repeated in triplicate for each strain. The values of the experimental replicates for each strain for each carbon and nitrogen substrate were compared by a one-way analysis of variance and Tukey *post hoc* multiple-comparison test, using a 95% family-wise confidence level. Statistical significance was assigned at the *P* ≤ 0.05 level.

### Bacterial growth in different carbon sources.

Bacteria were grown aerobically at 37°C for approximately 16 h in LB broth and then diluted to ~10^4^ CFU/ml in fresh M9 minimal medium. M9 minimal medium was used to coincide with that used in previous similar experiments (73). Growth kinetics were determined in M9 minimal medium supplemented with 6 mM l-histidine (Sigma-Aldrich, United Kingdom) (parental strain SL1344 is a histidine auxotroph) and added carbon source. Carbon sources tested were glucose (0.4%), fructose (0.4%), succinate (0.2%), aspartate (0.4%), pyruvate (0.2%), and saccharate (0.05%) (Sigma-Aldrich, United Kingdom). These concentrations were chosen based on preliminary experiments which determined the concentrations at which bacteria were able to grow. For glucose, fructose, and aspartate, absorbance was measured at 600 nm in a FLUOstar Optima apparatus (BMG Labtech, United Kingdom) at 37°C with agitation before each reading, which was taken every 5 min for 19 h. Data were collected from three biological and three technical replicates of each strain and are presented as the average optical density over time. For succinate, pyruvate, and saccharate, growth in the FLUOstar was poor, and therefore growth kinetics were determined by inoculating 10 ml M9 minimal medium with histidine and the specified carbon source. Cultures were incubated at 37°C with agitation, and absorbance at 600 nm (OD_600_) was measured initially and at 2, 4, 6, 8, 10, 12, and 24 h. Final density (24 h) and generation time during exponential phase were calculated and analyzed using a two-tailed Student’s *t* test. *P* values of ≤0.05 were considered significant.

### Anaerobic growth.

Cultures were set up in an anaerobic chamber (anaerobic gas growth mixture of 10% CO_2_, 10% H_2_, 80% N_2_) in MOPS minimal medium supplemented with glucose and 6 mM l-histidine and incubated at 37°C for 24 h. Growth curves in MOPS minimal medium with histidine were started with 4% inoculum from overnight cultures grown anaerobically, and the initial OD_600_ was measured. The OD_600_ was measured every 2 h for the first 10 h and again at 24 h. Three independent experiments with three biological replicates were completed. Data are presented as the mean generation time ± the standard deviation. *P* values of ≤0.05 from a two-tailed Student’s *t* test were considered significant.

### Motility assays.

Bacterial strains were grown overnight at 37°C in LB broth with or without antibiotic, as appropriate. LB broth was used because nutrient-rich broth is required for *Salmonella* swarming. To prepare inocula, cells were adjusted to an OD_600_ of 0.5 in phosphate-buffered saline (PBS). Motility plates were prepared (the night before inoculation to allow time to set) by combining LB broth and Difco Bacto agar (BD); for swimming assays, an agar concentration of 0.3% was used, and for swarming assays an agar concentration of 0.6% was used with the addition of 5 g/liter glucose. To inoculate swimming plates, a straight loop was immersed in the inoculum and then stabbed into the center of the swimming plate. These plates were then incubated for 7 h at 37°C. For swarming assays, 5-µl aliquots of inoculum were spotted on the top of swarming plates, which were incubated for 16 h at 37°C. Images were taken using a Syngene Transilluminator box, and ImageJ software was used to quantify the area covered. Data were analyzed using a two-tailed Student’s *t* test, and *P* values of ≤0.05 were considered significant.

### Infection of tissue culture cells.

Caco-2 epithelial cells were grown in Dulbecco’s minimal essential medium (DMEM; Sigma-Aldrich, United Kingdom) containing 1% (vol/vol) nonessential amino acids (Sigma-Aldrich, United Kingdom), 1% (vol/vol) l-glutamine (Sigma-Aldrich, United Kingdom), and 10% fetal bovine serum (FBS; Sigma-Aldrich, United Kingdom). Cells were polarized by seeding at 1.8 × 10^5^ cells per well and grown for 2 weeks, with fresh DMEM added every 2 to 3 days. Infection assays were carried out as described previously (52). Briefly, overnight cultures of SL1344 wild-type and the *acrD*::*aph* mutant were grown at 37°C in LB broth, washed in PBS, and then 1 × 10^7^ bacteria/ml was added to inoculation medium (DMEM without FBS). Caco-2 cells were washed 3 times with warm Hanks’ balanced salt solution (HBSS; Sigma-Aldrich, United Kingdom), and then 1 ml of inoculation medium was added to each well. At 2 h postinfection, all cells were washed with HBSS and for the 2-h time point cells, were lysed using 1% Triton X-100 (Sigma-Aldrich, United Kingdom), serially diluted, and plated on LB agar for bacterial enumeration. For later time points, after washing the inoculation medium containing 100 µg/ml of gentamicin was added to each well and incubated for another 2 h. For the 4-h time point, cells were lysed, serially diluted, and enumerated as described before. For the 8- and 24-h time points, medium was replaced with inoculation medium containing 10 µg/ml gentamicin and cultures were incubated for the remaining time. Cells were lysed, diluted, and enumerated as described above.

Competitive infections were carried out alongside isolated infections (described above), with the following changes. Inoculum was prepared by combining 5 × 10^6^ bacteria/ml of wild-type SL1344 and 5 × 10^6^ bacteria/ml of the mutant *acrD*::*aph* strain. Inocula and samples from each time point were serially diluted and plated on LB agar and incubated for 14 to 16 h at 30°C to ensure small colonies. Total colonies were enumerated. Using velvet squares, colonies were replica plated onto LB agar containing 50 µg/ml kanamycin and then incubated for 16 h, aerobically. Kanamycin-resistant colonies were enumerated.

Data are presented as the mean CFU of three independent experiments with four replicates each, ± standard deviations and were analyzed using a two-tailed Student’s *t* test. *P* values of ≤0.05 were considered significant.

### Competition assays.

Bacterial strains were grown statically at 37°C overnight in LB broth with or without antibiotic, as appropriate. Cells were washed in PBS, and the cultures were adjusted until the OD_600_ was 0.3. Wild-type SL1344 (100 µl) and mutant *acrD*::*aph* cells were added to 10 ml of LB broth (no antibiotics) and incubated at 37°C with aeration. Fresh broth was inoculated every 24 h. CFU were determined by serial dilution and plating on LB agar plates at 0 h (inoculum) and 2, 4, 6, 8, 10, 12, 24, 48, and 72 h. Replica plating with velvet squares onto LB agar plates containing 50 µg/ml of kanamycin was performed to determine the ratio of wild-type to mutant cells, and competition indices were determined. Three independent experiments were completed, with three biological replicates each. Data are presented as mean CFU ± standard deviations and analyzed using a two-tailed Student’s *t* test. *P* values of ≤0.05 were considered significant.

### SPI-1 reporter assays.

The chromosomal *gfp* reporter fused to the promoter of *prgH* has been previously described (31), and we have previously used *gfp* with the *prgH* promoter to measure activity of SPI-1 in individual cells (34). In brief, strains SL1344 *prgH′-gfp* and SL1344 *acrB*::*aph prgH′-gfp* were grown to mid-logarithmic phase in MOPS minimal medium. The cells were harvested by centrifugation at 2,200 × *g* at room temperature, washed, and resuspended in PBS. Bacteria were analyzed by flow cytometry using a FACSAria2 system (BD Biosciences). Cells were illuminated with a 488-nm laser, and scatter and GFP fluorescence data were collected through a 502 LP mirror and 530/30 bandpass filter. For each sample, 10,000 events were collected.

## SUPPLEMENTAL MATERIAL

Figure S1  Growth of SL1344 (blue line) and *acrD*::*aph* (red line) in M9 minimal medium supplemented with either glucose (0.4%), fructose (0.4%), succinate (0.2%), aspartate (0.4%), pyruvate (0.2%), or saccharate (0.05%). Download Figure S1, EPS file, 1 MB

Figure S2  Generation time and final optical density of *acrD*::*aph* and SL1344 cultures grown in LB broth. Data presented are the means of three experiments performed in triplicate ± standard deviations. No statistically significant differences were found between groups. Download Figure S2, EPS file, 0.6 MB

Figure S3  Growth of SL1344 and *acrD*::*aph* cultures grown in medium containing 6 different carbon sources. Growth in M9 minimal medium supplemented with glucose (0.4%), fructose (0.4%), succinate (0.2%), aspartate (0.4%), pyruvate (0.2%), or saccharate (0.05%) is shown. Data are the mean results of three independent experiments ± standard deviations. (a) Generation time of wild-type SL1344 (blue bars) and SL1344 *acrD*::*aph* (red bars) cultures grown in glucose, fructose, succinate, aspartate, pyruvate, or saccharate. (b) Final optical density of wild-type SL1344 (blue bars) and SL1344 *acrD*::*aph* (red bars) cultures grown in glucose, fructose, succinate, aspartate, pyruvate, or saccharate. No statistically significant differences were found between groups of data. Download Figure S3, EPS file, 0.9 MB

Figure S4  Anaerobic growth of SL1344 and *acrD*::*aph*. (a) Generation time of strains grown anaerobically in MOPS minimal medium. (b) Final optical density of strains grown anaerobically in MOPS minimal medium. Data presented are the mean results of three experiments performed in triplicate ± standard deviations. No statistically significant differences were found between data groups. Download Figure S4, EPS file, 0.6 MB

Figure S5  Invasion of polarized Caco-2 epithelial cells. Association was determined at 2 h postinfection, prior to gentamicin treatment (*P* = 0.745). A gentamicin protection assay was performed to determine invasion and persistence at 4, 8, and 24 h postinfection (*P* = 0.846, *P* = 0.942, and *P* = 0.685, respectively). Each data point is the mean results for one independent experiment comprising 4 replicates. Download Figure S5, EPS file, 0.6 MB

Table S1  Significant (adjusted *P* < 0.004) gene expression changes in *acrD*::*aph* cultures compared to the wild type (data are the fold change relative to growth of SL1344; boldface indicates increased gene expression and italics indicate decreased gene expression). Table S1, DOCX file, 0.02 MB
