# Supplementary material for: Beyond Antimicrobial Resistance: Evidence for a Distinct Role of the AcrD Efflux Pump in Salmonella Biology
Source: mBio. 2016 Nov 22;7(6):e01916-16. doi: 10.1128/mBio.01916-16 (PMC5120143; doi:10.1128/mBio.01916-16)
Supplement: Table S1 — Significant (adjusted P < 0.004) gene expression changes in acrD::aph cultures compared to the wild type (data are the fold change relative to growth of SL1344; boldface indicates increased gene expression and italics indicate decreased gene expression). [file mbo006163077st1.docx]

**Table S1. Significant (adjusted p < 0.004) gene expression changes in *acrD*::*aph*.** Data in fold change relative to SL1344. **Bold** indicates increased gene expression; *italics* indicate decreased gene expression.

| **Gene Name** | **Product** | **Fold Change** |
| --- | --- | --- |
| **Pathogenicity** |  |  |
| *cigR* | putative exported protein | *0.55* |
| *orgA* | oxygen-regulated invasion protein | **1.81** |
| *sicP* | chaperone (associated with virulence) | *0.53* |
| *sscA* | putative Type III secretion system chaperone protein | *0.44* |
| *sscB* | putative pathogenicity island protein | *0.22* |
| *sseB* | putative pathogenicity island effector effector protein | *0.27* |
| *sseC* | putative pathogenicity island effector protein | *0.36* |
| *sseD* | putative pathogenicity island effector protein | *0.21* |
| *sseE* | putative pathogenicity island effector protein | *0.28* |
| STM1025 | putative lipoprotein | **3.42** |
| STM1389 | putative pathogenicity island protein | *0.47* |
| STM1410 | putative pathogenicity island protein | *0.45* |
| STM2904 | hypothetical protein | *0.2* |
| STM2905 | hypothetical protein | *0.22* |
| STY2029 | putative bacteriophage protein | *0.05* |
| **Sigma factors** |  |  |
| *rpoD* | RNA polymerase sigma-70 factor | *0.74* |
| *rpoS* | RNA polymerase sigma subunit (sigma-38) | *0.27* |
| **Stress response** | |  |
| *cspA* | cold shock protein | **11.24** |
| *cspD* | cold shock-like protein | *0.31* |
| *cstA* | carbon starvation protein A | *0.37* |
| *dnaK* | DnaK protein (heat shock protein 70) | *0.27* |
| **Amino acid metabolism** | | |
| *argD* | acetylornithine aminotransferase | **3.42** |
| *asd* | aspartate-semialdehyde dehydrogenase | **1.75** |
| *aspA* | aspartate ammonia-a | **10.19** |
| *dapA* | dihydrodipicolinate synthase | **1.29** |
| *glnA* | glutamine synthetase | **3.57** |
| *hemB* | delta-aminolevulinic acid dehydratase | **1.91** |
| *mgsA* | methylglyoxal synthase | **2.23** |
| *purA* | adenylosuccinate synthetase | **2.23** |
| *speE* | spermidine synthase | **3.06** |
| *oat* | putrescine aminotransferase | *0.18* |
| *yhbS* | putative acetyltransferase | **1.57** |
| *tdh* | threonine 3-dehydrogenase | *0.43* |
| *thrC* | threonine synthase | **2.56** |
| *ybiK* | putative L-asparaginase | *0.32* |
| **Anaerobic respiration** | |  |
| *napA* | probable nitrate reductase | **3.54** |
| *napC* | cytochrome c-type protein | **7.85** |
| *napF* | ferredoxin-type protein | **3.06** |
| *nirB* | nitrite reductase large subunit | **10.48** |
| *nirC* | putative nitrite transporter | **21.19** |
| *nirD* | nitrite reductase (NAD(P)H) small subunit | **22.83** |
| **ATP Synthesis** | |  |
| *atpA* | ATP synthase alpha subunit | **1.73** |
| *atpC* | ATP synthase epsilon subunit | **1.78** |
| *atpD* | ATP synthase beta subunit | **2.3** |
| *atpE* | ATP synthase subunit C | **1.75** |
| *atpF* | ATP synthase subunit B | **1.55** |
| *atpG* | ATP synthase gamma subunit | **1.84** |
| **Citrate/TCA Cycle** | |  |
| *acnA* | aconitate hydratase 1 (citrate hydro-lyase 1) | *0.2* |
| *acnB* | aconitate hydratase 2 (citrate hydro-lyase 2) | *0.56* |
| *frdA* | fumarate reductase, flavoprotein subunit | **8.41** |
| *frdB* | fumarate reductase, iron-sulfur protein | **7.66** |
| *frdC* | fumarate reductase complex subunit C; membrane anchor polypeptide | **5.12** |
| *frdD* | fumarate reductase complex subunit D; membrane anchor polypeptide | **5.74** |
| *fumC* | fumarate hydratase class II | *0.22* |
| *gdhA* | NADP-specific glutamate dehydrogenase | **1.62** |
| *gltA* | citrate synthase | *0.34* |
| *icdA* | isocitrate dehydrogenase | *0.38* |
| *maeB* | NADP-dependent malate dehydrogenase (decarboxylating) | *0.67* |
| *mdh* | malate dehydrogenase | *0.22* |
| *ppsA* | phosphoenolpyruvate synthase | *0.18* |
| *sdhA* | succinate dehydrogenase flavoprotein subunit | *0.27* |
| *sdhC* | succinate dehydrogenase cytochrome b-556 subunit | *0.31* |
| *sdhD* | succinate dehydrogenase hydrophobic membrane anchor protein | *0.39* |
| *sucA* | 2-oxoglutarate dehydrogenase E1 component | *0.63* |
| **Glycolysis/Gluconeogenesis** | | |
| *adh* | alcohol dehydrogenase | **70.39** |
| *eno* | Enolase | **2.37** |
| *fba* | fructose 1,6-bisphosphate aldolase | **3.58** |
| *fbp* | fructose-1,6-bisphosphatase | *0.53* |
| *gapA* | glyceraldehyde 3-phosphate dehydrogenase A | **2.46** |
| *lpdA* | dihydrolipoamide dehydrogenase | *0.4* |
| *pfkA* | 6-phosphofructokinase | **5.17** |
| *pflB* | formate acetyltransferase 1 | **11.36** |
| *pgi* | glucose-6-phosphate isomerase | **1.56** |
| *pgk* | phosphoglycerate kinase | **3.52** |
| *ppc* | phosphoenolpyruvate carboxylase | **2.24** |
| *pykA* | pyruvate kinase A | **1.82** |
| *rfbF* | glucose-1-phosphate cytidylyltransferase | **2.08** |
| *tpiA* | triosephosphate isomerase | **2.01** |
| *zwf* | glucose 6-phosphate dehydrogenase | *0.64* |
| **Purine Metabolism** | | |
| *cysA* | sulphate transport ATP-binding protein CysA | *0.09* |
| *cysC* | adenosine 5-phosphosulfate kinase | *0.13* |
| *cysD* | ATP sulfurylase (ATP:sulfate adenyltransferase) | *0.11* |
| *cysH* | 3-phosphoadenosine 5-phosphosulfate sulfotransferase | *0.1* |
| *cysI* | sulfite reductase (NADPH) hemoprotein alpha subunit | *0.17* |
| *cysJ* | sulfite reductase (NADPH) flavoprotein beta subunit | *0.26* |
| *cysK* | cysteine synthase A | *0.22* |
| *cysN* | ATP sulfurylase (ATP:sulfate adenyltransferase) subunit | *0.11* |
| *cysP* | thiosulphate-binding protein precursor | *0.29* |
| *cysU* | sulphate transport system permease protein CysT | *0.3* |
| *dgt* | deoxyguanosinetriphosphate triphosphohydrolase | *0.56* |
| *glyA* | serine hydroxymethyltransferase | *0.51* |
| *gppA* | guanosine-5-triphosphate,3-diphosphate pyrophosphatase | **2.92** |
| *guaC* | GMP reductase | *0.5* |
| *pnp* | polynucleotide phosphorylase | **2.05** |
| **Ribsomal biosynthesis subunit** | | |
| *rpoA* | DNA-directed RNA polymerase alpha chain | **2.26** |
| *rpoB* | DNA-directed RNA polymerase, beta-subunit | **2.14** |
| *rpoC* | DNA-directed RNA polymerase, beta-subunit | **1.68** |
| *rpsA* | 30S ribosomal protein S1 | **1.91** |
| *rpsI* | 30S ribosomal subunit protein S9 | **2.01** |
| *rpsK* | 30S ribosomal subunit protein S11 | **1.74** |
| *rpsL* | 30S ribosomal subunit protein S12 | **2.34** |
| *rpsM* | 30S ribosomal subunit protein S13 | **1.8** |
| *rpsP* | 30S ribosomal subunit protein S16 | **3.15** |
| **RNA polymerase** | |  |
| *rpoA* | DNA-directed RNA polymerase alpha chain | **2.26** |
| *rpoB* | DNA-directed RNA polymerase, beta-subunit | **2.14** |
| *rpoC* | DNA-directed RNA polymerase, beta-subunit | **1.68** |
| **Sugar transport** | |  |
| *fruA* | PTS system, fructose-specific IIBC component | **12.05** |
| *fruB* | pts system, fructose-specific IIA/FPR component | **45.02** |
| *fruK* | 1-phosphofructokinase | **2.92** |
| *manY* | phosphotransferase enzyme II, C component | **4.09** |
| *rfbK* | Phosphomannomutase | **2** |
| *rfbM2* | mannose-1-phosphate guanylyltransferase | **2.45** |
| *ptsG* | PTS system, glucose-specific IIBC component | **4.11** |
| *ptsN* | nitrogen regulatory IIA protein | *0.63* |
| **Ubiquinone Biosynthesis/Oxidative Phosphorylation** | |  |
| *nuoA* | NADH dehydrogenase I chain A | **2.16** |
| *nuoB* | NADH dehydrogenase I chain B | **2.04** |
| *nuoC* | NADH dehydrogenase I chain C; chain D | **1.66** |
| *nuoG* | NADH dehydrogenase I chain G | **1.92** |
| *nuoH* | NADH dehydrogenase I chain H | **1.71** |
| *nuoL* | NADH dehydrogenase I chain L | **1.73** |
